# Hepatitis C virus NS4 protein impairs the Th1 polarization of immature dendritic cells

**DOI:** 10.1111/j.1365-2893.2009.01213.x

**Published:** 2010-08

**Authors:** A Takaki, M Tatsukawa, Y Iwasaki, K Koike, Y Noguchi, H Shiraha, K Sakaguchi, E Nakayama, K Yamamoto

**Affiliations:** 1 Departments of Gastroenterology and Hepatology, Okayama University Graduate School of Medicine, Dentistry and Pharmaceutical SciencesOkayama, Japan; 2 Departments of Immunology, Okayama University Graduate School of Medicine, Dentistry and Pharmaceutical SciencesOkayama, Japan

**Keywords:** dendritic cell, hepatitis C virus, T cell

## Abstract

Dendritic cells (DCs) in chronic hepatitis C patients display impaired function, although the details remain unclear. To investigate the hepatitis C virus (HCV) protein that has the most impact on DC function, we compared five recombinant proteins and seven HCV protein genes in modulating DC phenotype and function. Immature DCs (iDCs) were established from healthy donor peripheral blood monocytes with granulocyte–macrophage colony stimulating factor (GM-CSF) and IL-4. Lipopolysaccharide was used to establish mature DCs (mDCs). Cells were then pulsed with HCV recombinant proteins or transfected with HCV plasmids and subsequently assayed for cell surface marker expression by flow cytometry. For cytokine and proliferative T-cell response analysis, DCs were cultured with autologous CD4 T cells and tuberculin purified protein derivative (PPD). Mean fluorescent intensity of CD86 was reduced in HCV protein-pulsed iDCs. Proliferative T-cell responses and Th1 cytokine concentrations were reduced with HCV nonstructural proteins (NS), particularly with HCV NS4. HCV nonstructural proteins, particularly NS4, change the iDC phenotype and reduce antigen-specific T-cell stimulatory function with Th1 cytokine reductions.

## Introduction

Hepatitis C virus (HCV) is a major causative agent of chronic hepatitis. About 70% of infected patients develop chronic hepatitis, subsequently resulting in liver cirrhosis and hepatocellular carcinoma [1]. Because HCV is not a cytopathic virus, immune reactions play a central role in the development of chronic hepatitis [2]. The lack of a strong Th1-type helper T-cell response and cytotoxic T-cell response against HCV leads to chronic infection with this virus [3–5]. Strong T-cell responses correlate with spontaneous recovery [3,6,7], but these responses are not correlated with interferon-induced sustained virological response (SVR) [8]. To identify mechanisms for these T-cell immune response defects in chronic hepatitis C, dendritic cells (DCs) function has been investigated in recent years.

DCs are thought to play a central role in the interplay between innate and adoptive immune responses. Recently, two subsets of DCs have been characterized. While myeloid DCs (mDCs) produce large amounts of IL-12 upon stimulation, plasmacytoid DCs (pDCs) produce large amounts of interferon-alpha (IFNα) in viral infection [9].

DC function has been reported as broadly impaired in chronic hepatitis C patients [10–13]. However, several reports have produced contradictory results that DC function is not impaired in chronic hepatitis C patients [14–18]. Most of these reports are studies employing mDCs. However, pDCs are also reportedly functionally impaired and reduced in number by increased apoptosis [19]. Because culture conditions and chronic hepatitis conditions in the patient may change the phenotype of immune cells, functional differences in DCs during chronic HCV infection remain contentious. *In vitro* transfection or addition of HCV proteins such as core or NS3 has been reported to result in reduced function of DCs. Because of constrains in maintaining long term *in vitro* culture systems for HCV, these experimental results are also contentious. Recently, Shiina *et al.* [9] have shown that using an infectious cell-culture produced HCV impaired pDC function that could be more likely the *in vivo* condition than former experiments using HCV plasmids or recombinant proteins suggested.

HCV possesses a single-strand, positive-sense RNA genome of approximately 9.6 kb encoding a single polyprotein [20]. This precursor protein is cleaved into at least 10 proteins. These proteins reportedly display many functions that are related to the host immune system. The core protein reportedly inhibits differentiation and allo- and auto-stimulatory functions of DCs and may induce interleukin (IL)-10 over-production [13]. The NS3 protein binds directly to interferon regulatory factor (IRF) 3 to downregulate its function [21]. The NS4 protein slows the rate of endoplasmic reticulum (ER)-to-Golgi traffic, interfering with maturation of the HLA complex [22]. NS5a downregulates host interferon responses against viral infection [23]. As a result, almost all HCV component proteins exhibit functionality in interfering with host immune pressure. However, which protein exerts the most effect on escaping from immune pressure remains unclear.

The present study examined the effect of individual HCV proteins (core, NS2, NS3, NS4 and NS5) on DCs function from healthy volunteers in relation to auto-stimulation of CD4 T cells. We found that HCV NS4 had the most effect in downregulating Th1-type helper T-cell responses in comparison with other HCV proteins.

## Materials and methods

### Samples

Subjects comprised of 8 healthy male volunteers (serologically negative for HCV, hepatitis B virus and human immunodeficiency virus, mean age, 33 ± 5 years). All study protocols were approved by the Committee for Human Subjects in Research at Okayama University, and informed consent was obtained from all participants.

### HCV recombinant proteins and HCV-containing plasmids

HCV recombinant proteins including core, NS3, NS4, NS5A and NS5B were purchased from Mikrogen GmbH (Martinsried, Germany), respectively. Mammalian expression plasmids pCXN2-core, -NS2, -NS3, -NS4A, -NS4B, -NS5A and -NS5B, which contained the respective HCV genomic regions and are driven by a β-actin-based CAG promoter [24], were kindly donated by Dr Naoya Kato from the Department of Gastroenterology at Tokyo University.

### CD14-positive monocyte isolation and myeloid DC generation

Mononuclear cells were separated from peripheral blood by centrifugation on Ficoll–Hypaque density gradient (Amersham Pharmacia, Uppsala, Sweden), as previously described. CD14-positive monocytes were purified positively using microbeads (Miltenyi Biotec, Auburn, CA, USA) in accordance with the protocols of the manufacturer. In brief, peripheral blood mononuclear cells (PBMCs) were incubated with CD14 microbeads, and then CD14-positive cells were positively sorted using a magnetic-activated cell-sorting system (MACS; Miltenyi Biotec). The positively selected fraction proved to be >95% positive for CD14 by flow cytometric analysis staining with FITC-conjugated anti-CD14 antibody (BD Pharmingen, San Jose, CA). CD14-positive cells were cultured at 1 × 10^6^mL−1 in RPMI supplemented with 5% heat-inactivated human AB serum (ICN Biomedicals; Aurora, OH, USA), 100 ng mL−1 of granulocyte–macrophage colony stimulating factor (GM-CSF) (kindly provided by Kirin Pharma, Tokyo, Japan) and 50 ng mL−1 of IL-4 (kindly provided by Ono Pharmaceuticals, Osaka, Japan) at 37 °C in 5% CO_2_ for 5 days. Cells proved to be CD11c-positive myeloid immature DCs (iDCs).

Cultures were pulsed with 1 μg mL−1 of each HCV protein. For transfection experiments, DCs were transfected with HCV plasmids by lipofection using Effectene transfection reagent (Qiagen, San Jose, CA, USA) according to the manufacturer’s protocols. HCV protein-pulsed and HCV-transfected DCs were cultured for an additional 1 day and then used in the following experiment. For maturation using mature DCs (mDCs), 1 ng mL−1 of lipopolysaccharide (LPS) (Sigma, St Louis, MO, USA) was added to the culture 1 day after HCV protein addition or HCV plasmid transfection.

### DC surface marker analysis by flow cytometry

DCs were stained with FITC-conjugated monoclonal antibodies (anti-human CD11c, CD40, CD86, HLA-ABC, HLA-DR and relevant isotype controls; BD Pharmingen, San Diego, CA). Cells (1 × 10^5^) were incubated with each antibody for 20 min at 4 °C in the dark and washed twice with 1% FCS PBS. Cells were analysed using FACScan (BD Immunocytometry Systems, San Diego, CA, USA). Expression of each surface marker was analysed by CellQuest (BD Immunocytometry Systems). Mean fluorescent intensity (MFI) of each surface marker was calculated, and results were expressed as MFI index (MFI of the target protein/MFI of buffer control or control plasmid).

### Quantification of CD4 T-cell stimulatory function

HCV recombinant protein-pulsed DCs or HCV plasmid-transfected DCs were tested for antigen-specific autologous-CD4 T-cell stimulatory function. DCs (3 × 10^3^ per well) were cultured with auto-CD4 T cells (2 × 10^5^ per well) as responder cells and with tuberculin purified protein derivative (PPD) (Japan BCG Laboratory, Tokyo, Japan) as a recall antigen in 96-well round bottom plates (#3799; Corning, NY) at a concentration of 0.1 μg mL−1. Next, [*methyl*-^3^H]-thymidine (TRK637; GE Healthcare Amersham Biosciences, Buckinghamshire, UK) was added on day 5, and thymidine incorporation was measured after 16 h. Results were recorded as relative response index (cpm of the target protein/cpm of buffer control or control plasmid).

### Cytokine analysis

Supernatants of recombinant protein-pulsed or plasmid-transfected DCs cocultured with CD4 T cells (2 × 10^5^ well-1), and PPD (0.1 μg mL−1) were collected on day 2 and stored at −80 °C. Supernatants were thawed just before use and analysed for interferon (IFN)-γ, IL-2, IL-4 and IL-6 production using a particle-based immunoassay that detects amplified fluorescence by flow cytometry (BD Cytometric Bead Array Human Th1 Th2-1 Cytokine Kit-II; BD Biosciences Pharmingen, San Diego, CA, USA). Results were recorded as relative concentration index (pg mL−1 of target protein/pg mL−1 of buffer control or control plasmid).

### Statistical analysis

Results were expressed as relative indices compared to control. To compare the mean values of relative indices, the Kruskal–Wallis test with the Dunnett post-test or Student’s *t*-test was used with JMP software (SAS Institute, Cary, NC, USA).

## Results

### Effect of recombinant HCV protein addition on surface marker expression of DCs

**Fig. 1:**
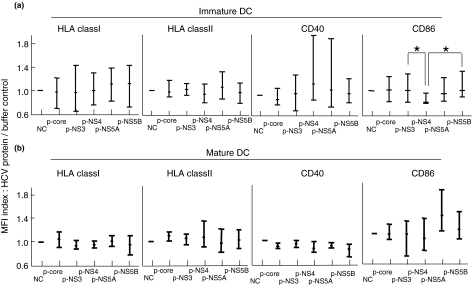
MFI of HLA and co-stimulatory molecules on dendritic cells (DCs) was reduced with hepatitis C virus (HCV) protein addition. HCV protein-pulsed DCs are shown as p-core, p-NS3, p-NS4, p-NS5A, p-NS5B. (a) MFI index of HLA class I and II and co-stimulatory molecules CD40 and CD86 expressed on HCV protein-pulsed DCs. CD86 was reduced by addition of HCV NS4 protein compared with HCV NS3 and NS5B. (b) MFI of surface markers of DCs pulsed with HCV recombinant proteins after maturation with LPS. HCV protein-pulsed DCs showed comparable ability to mature in terms of surface expression of co-stimulatory molecule. *P < 0.05 (Student’s *t-*test).

Mean ratios of surface markers on DCs following addition of HCV recombinant proteins for the eight donors are shown in Fig. 1. The expression of CD86 was reduced in DCs exposed to NS4 compared with NS3 or NS5B (Fig. 1a). However, this difference was overcome with maturation of DCs (Fig. 1b).

### Effect of HCV protein encoding plasmid transduction on DC surface marker expression

**Fig. 2:**
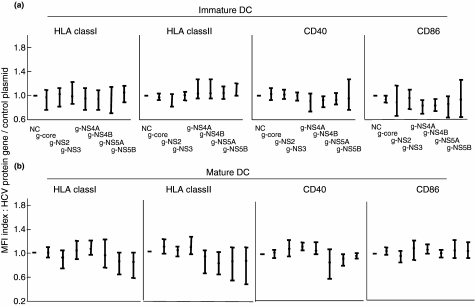
MFI of HLA class I and II and co-stimulatory molecules CD40 and CD86 expressed on dendritic cells (DCs) transfected with hepatitis C virus (HCV) protein encoding plasmids. HCV protein encoding plasmids are shown as g-core, g-NS2, g-NS3, g-NS4A, g-NS4B, g-NS5A, g-NS5B. (a) HCV protein encoding plasmid-transduced DCs showed comparable ability to mature in relation to surface expression of co-stimulatory molecules. (b) MFI of surface markers on DCs matured with LPS following transfection with HCV protein encoding plasmids. These DCs showed comparable ability to mature in terms of surface expression of co-stimulatory molecules.

MFI ratios of surface markers on DCs following transduction with HCV protein encoding plasmids are shown in Fig. 2. CD86 expression once again was relatively reduced with HCV NS4A (Fig. 2a), although this did not reach statistical significance.

However, maturation of these iDCs with LPS overcame the reduced CD86 expression induced by HCV-plasmid transfection (Fig. 2b).

### The effect of recombinant HCV proteins and HCV plasmids on PPD-specific CD4 T-cell stimulatory function of DCs

**Fig. 3:**
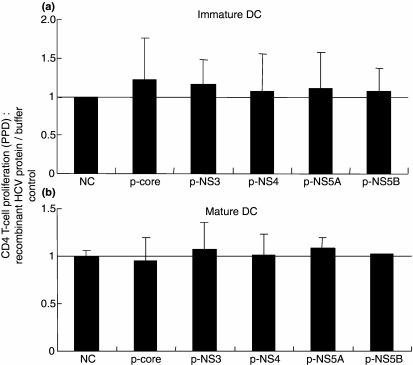
CD4 T-cell proliferation with tuberculin purified protein derivative (PPD) and dendritic cells (DCs) was not affected by recombinant hepatitis C virus (HCV) protein addition. (a) CD4 T-cell proliferation with PPD and DCs after addition of HCV core, NS3, NS4, NS5A and NS5B recombinant proteins. No difference was observed in PPD-specific T-cell proliferative responses with HCV recombinant protein addition. (b) DCs pulsed with HCV recombinant proteins and subsequent maturation with LPS. As in the case of iDCs, no difference was observed in PPD-specific T-cell proliferative responses with HCV recombinant protein addition.

**Fig. 4:**
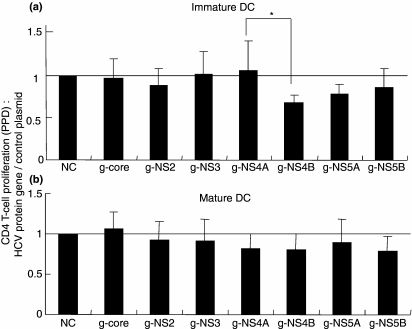
Effect on CD4 T-cell proliferation with tuberculin purified protein derivative (PPD) following transfection with hepatitis C virus (HCV) protein encoding plasmids. (a) CD4 T-cell proliferation with PPD and iDCs was relatively reduced by exposure to NS4B compared with NS4A. (b) Dendritic cells (DCs) transduced with HCV plasmids and subsequently matured with LPS. Relative reduction in CD4 T-cell stimulatory function by NS4B was overcome by LPS maturation. *P < 0.05 (Student’s *t-*test).

To investigate the effect of HCV on antigen-specific immune responses, a recall antigen PPD was used as a stimulant for DC and auto-CD4 T-cell co-culture experiments. HCV protein-pulsed DCs showed no difference in T-cell stimulatory responses (Fig. 3). HCV plasmid transfection on the other hand showed low stimulatory responses with NS4B compared with NS4A (Fig. 4a).

Maturation of DCs with LPS overcame the impaired PPD-specific CD4 T-cell stimulatory function of DCs (Fig. 4b).

### Regulatory effect of recombinant HCV proteins on Th1 cytokine production in PPD, CD4 T cell and DC co-culture system

**Fig. 5:**
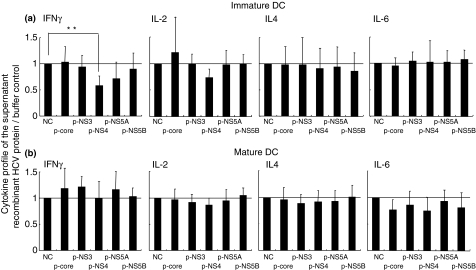
Recombinant NS4 protein addition reduced production of Th1 cytokines in antigen-specific CD4 T-cell responses. (a) Cytokine profile of supernatant from mixed culture of CD4 T cells, PPD and dendritic cells (DCs) after pulsing with hepatitis C virus (HCV) recombinant proteins. NS4 addition reduced production of Th1 cytokines IFNγ and IL-2, whilst Th2 cytokines IL4 and IL6 were unaffected. (b) Maturation of DCs with LPS rescued Th1 cytokine production after pulsing with HCV recombinant proteins. No effect was seen with HCV protein encoding plasmids. *P < 0.05 (Dunnett post-test).

Supernatant from CD4 T cells and DC co-cultures was collected and analysed for Th1 and Th2 cytokine production. IFNγ and IL-2, as representative Th1 cytokines, were decreased with addition of NS4 (Fig. 5a). Conversely, IL-4 and IL-6, as representative Th2 cytokines, were unaffected by HCV protein addition.

Maturation of DCs with LPS once again overcame the impaired PPD-specific CD4 T-cell stimulatory function of DCs (Fig. 5b).

**Fig. 6:**
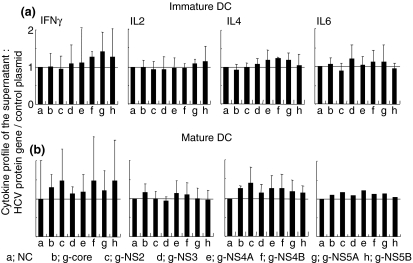
Cytokine profile in supernatants from mixed cultures of CD4 T cells, tuberculin purified protein derivative (PPD) and dendritic cells (DCs) transduced with hepatitis C virus (HCV) protein encoding plasmids. Maturation of DCs with LPS rescued the Th1 cytokine production. (a) Cytokine profile in supernatants from mixed cultures of CD4 T cells, PPD and iDCs transduced with HCV protein encoding plasmids. Transduction produced no effect. (b) Cytokine profile in supernatant from mixed cultures of CD4 T cells, PPD and DCs transduced with HCV protein encoding plasmids and subsequently matured with LPS (mDCs). As in the case of iDCs, no effect was seen with HCV plasmid-transfected DCs.

The same experiment was performed with plasmids encoding HCV proteins following transfection of DCs. Production of Th1 and Th2 cytokines was unaffected by HCV plasmid transduction (Fig. 6).

## Discussion

Our results demonstrate that HCV recombinant protein pulsing or HCV plasmid transfection of myeloid DCs reduces expression of co-stimulatory molecules and reduces Th1 immune responses. However, maturation of DCs overcame HCV-induced deterioration of the myeloid DC phenotype and restored function.

DCs are the most potent antigen-presenting cells that can activate naïve CD4 T cells. While isolating a sufficient number of native circulating DCs from PBMCs for functional analysis is difficult, myeloid DCs differentiated from peripheral blood monocytes can be used by culturing with GM-CSF and IL-4. In patients with chronic hepatitis C, some studies have proposed a defect in myeloid DC function [10–13], while others have found no such defect [14–18]. These data were derived using immunological assays of peripheral blood lymphocytes from chronic hepatitis C patients. These assay results could be influenced by the grade and stage of chronic hepatitis or co-existing clinically negligible diseases such as atopic dermatitis, allergic pharyngitis and the common cold, all of which may change the function of immune cells. Serum autoantibodies are more common in chronic hepatitis C patients with severe biochemical activities and histological grading [25] and are also significantly associated with cirrhosis and older age [26]. Hypergammaglobulinemia is another typical finding in liver cirrhosis patients. In such conditions showing autoimmune phenomena, B cells and related immune cells such as DCs and helper T cells should be more activated and more Th2-polarized than in patients without autoimmune phenomena.

The most frequently used immunological assay in previous studies was allo-stimulatory function analysis using allogenic mixed lymphocyte reactions (MLR) [10,11,13–17]. The MLR is a mixed culture response with DCs of chronic hepatitis C patients and T cells of healthy donors. This response depends on the degree of HLA class II matching between donors [27]. Because HLA class II haplotype reportedly influences outcome in chronic hepatitis C patients [28–30], HLA class II distribution may differ between chronic hepatitis C patients and healthy donors. In such a case, MLR responses would be influenced by different HLA class II distributions in chronic hepatitis C patients.

To overcome the problems associated with allo-MLR assays using patient lymphocytes and to evaluate the influence of HCV on DC function, we compared the phenotype and autologous CD4 T-cell stimulating function of myeloid iDCs established from healthy donor peripheral blood with HCV recombinant protein pulses and HCV protein encoding plasmids following transfection. As a recall antigen, we used PPD to stimulate DCs and autologous CD4 T cells in a mix culture that is unaffected by HLA class II distribution in the study population. In Japan, the Bacille Calmette Guerin (BCG) vaccination has been enforced since 1951, because of the nationwide tuberculosis endemic after World War II [31]. PPD is thus a good candidate for a recall antigen to detect antigen-specific immune responses in the Japanese population.

In the present study, functional experiments were all planned using healthy donor autologous-lymphocytes that should not be affected by HLA matching. HCV recombinant proteins and HCV plasmids, particularly NS4, reduced auto-stimulatory functions. Antigen-specific CD4 T-cell stimulatory function of DCs was evaluated using this autologous CD4 T cell and DC co-culture with PPD. Recombinant HCV protein addition revealed that NS4 protein showed the strongest reduction in CD86 expression on iDCs and Th1 cytokine production. The transduction experiment also showed that NS4 protein relatively impaired the antigen-presenting function.

Patients infected with human immunodeficiency virus (HIV) show clinical immune defects and also *in vitro* functional defects of immune cells such as DCs in addition to CD4 T cells that are infected with the virus [32,33]. Although HCV-infected patients might show reduced function of DCs, they are not clinically immunosuppressed even after several decades of infection. Our results indicate that HCV proteins cause a relative reduction in surface expression of the important co-stimulatory molecule CD86 on iDCs, but LPS maturation can rescue this effect. This may explain the clinical observation that HCV-infected individuals are not overtly immunosuppressed as HIV- infected patients.

NS4 protein is a required cofactor for NS3 protease activity. Co-immunoprecipitation experiments have shown that NS4A and NS4B are found in membrane-associated complexes [34]. NS4B is an integral membrane protein, mostly localized on the cytoplasmic side of the endoplasmic reticulum (ER) [35]. The precursor protein NS4A/B impedes ER-to-Golgi traffic, thus reducing the rate of appearance of HLA class I on the cell surface [22]. NS4A and NS4B inhibit cellular protein synthesis by targeting the process of translation [36], reducing immunological pressure from the infected individual. These previously reported functions of NS4 protein might explain our results of the effect of NS4 on DC function.

Th1 and Th2 polarization would decide the clinical course. Strong Th1 responses may eliminate HCV following spontaneous resolution, but weak Th1 and strong Th2 responses may result in chronic hepatitis. The present results show that addition of HCV recombinant proteins to iDCs results in weakened Th1 cytokine production without affecting Th2 production. This may explain the high chronicity rate of HCV. LPS maturation recovered this Th1 defect and may explain the immunologically competent function of chronic hepatitis C patients.

In conclusion, our study shows that HCV proteins, particularly NS4, affect the phenotype and function of iDCs, but phenotype and function recover after maturation with LPS. This is consistent with the observation that HCV is difficult to eliminate, even though HCV-infected patients show no signs of impaired immune response.

## Abbreviations:

BCG: Bacille Calmette Guerin
DC: Dendritic cell
ER: endoplasmic reticulum
GM-CSF: granulocyte–macrophage colony stimulating factor
HCV: hepatitis C virus
HIV: Human immunodeficiency virus
IFN: interferon
IRF: interferon regulatory factor
LPS: lipopolysaccharide
MFI: Mean fluorescent intensity
MLR: mixed lymphocyte reactions
PBMCs: peripheral blood mononuclear cells
pDC: plasmacytoid dendritic cells
PPD: tuberculin purified protein derivative
SVR: sustained virological response
